# Single-Subject Research in Psychiatry: Facts and Fictions

**DOI:** 10.3389/fpsyt.2020.539777

**Published:** 2020-11-13

**Authors:** Marij Zuidersma, Harriëtte Riese, Evelien Snippe, Sanne H. Booij, Marieke Wichers, Elisabeth H. Bos

**Affiliations:** ^1^Department of Psychiatry, Interdisciplinary Center Psychopathology and Emotion Regulation, University Medical Center Groningen, University of Groningen, Groningen, Netherlands; ^2^Department of Developmental Psychology, Faculty of Behavioural and Social Sciences, University of Groningen, Groningen, Netherlands

**Keywords:** psychiatry, single-subject, idiographic, nomothetic, N-of-1, intra-individual

## Abstract

Scientific evidence in the field of psychiatry is mainly derived from group-based (“nomothetic”) studies that yield group-aggregated results, while often the need is to answer questions that apply to individuals. Particularly in the presence of great inter-individual differences and temporal complexities, information at the individual-person level may be valuable for personalized treatment decisions, individual predictions and diagnostics. The single-subject study design can be used to make inferences about individual persons. Yet, the single-subject study is not often used in the field of psychiatry. We believe that this is because of a lack of awareness of its value rather than a lack of usefulness or feasibility. In the present paper, we aimed to resolve some common misconceptions and beliefs about single-subject studies by discussing some commonly heard “facts and fictions.” We also discuss some situations in which the single-subject study is more or less appropriate, and the potential of combining single-subject and group-based study designs into one study. While not intending to plea for single-subject studies at the expense of group-based studies, we hope to increase awareness of the value of single-subject research by informing the reader about several aspects of this design, resolving misunderstanding, and providing references for further reading.

## Introduction

Scientific evidence in the field of psychiatry mainly relies on studies that evaluate what is true on average in the population or a group. In many instances these studies yield valuable information, but particularly when the goal is to improve patient care we need to answer questions that apply to individual patients. For instance, if we want to know whether an antidepressant drug is effective in a particular patient, it will not suffice to know that this drug results in an average reduction of 0.31 SD in depressive symptoms in the population (1). Also, knowing that at the group level depressive symptoms are associated with increased levels of inflammatory markers (2) will not inform us whether for a specific patient depressive symptoms will increase when levels of inflammatory markers increase. It is increasingly being recognized that there are great inter-individual differences in causes, risk factors, and course over time of psychiatric disorders and their symptoms, and their response to treatments [e.g., (3, 4)].

**Figure 1:**
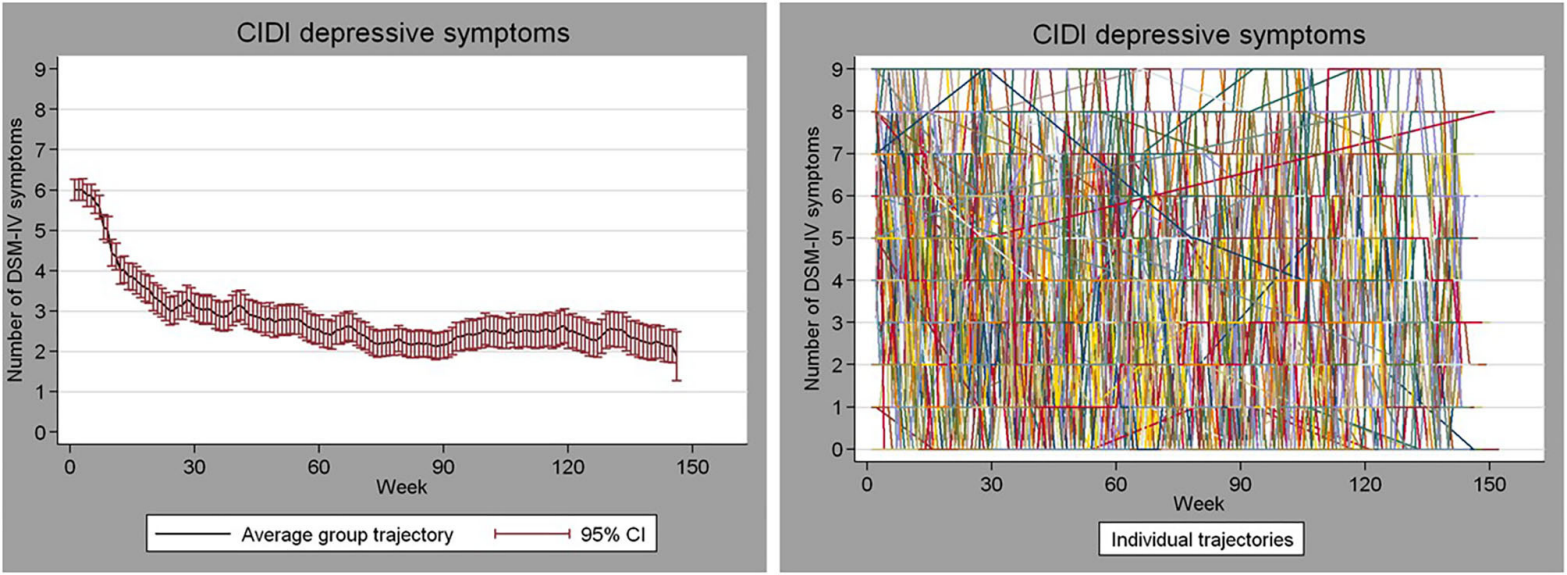
Weekly assessed depressive symptom severity over a period of 3 years in 267 persons who were initially depressed. **Left**: mean (95% CI) symptom severity. **Right**: trajectories of the individual persons.

To illustrate the potential magnitude of this heterogeneity, Figure 1 shows the course over time of depressive symptoms weekly assessed over a period of 3 years in 267 persons who were depressed at baseline [see for study details (5)]. The Figure shows that there are great differences between persons in the trajectories and that most persons show substantial fluctuations in symptom levels over time. It seems that for very few persons the average trajectory (left panel) applies, even were it to some extent. So, one may wonder to what extent such group-level results will give us information about what happens in most individual persons studied in that group.

What the Figure shows is not an extraordinary pattern, and many authors have noted the problem of relying on averages when no subject is average [e.g., (6–12)]. Many phenomena we study in the field of psychiatry are very heterogeneous across people, and most phenomena are not static but are highly dynamic (for example mood regulation and stress physiology). In the presence of such great inter- and intra-individual variability, information at the individual-person level may be of great value for making personalized treatment decisions or identifying personal predictors of changes in symptoms. Furthermore, in order to grasp the highly dynamic nature of certain phenomena we would need multiple repeated assessments across time. The single-subject study is a useful study design that can be used to make inferences about individual persons and to uncover the highly dynamic nature of our variables of interest. Nevertheless, this design is rarely used in the field of psychiatry. We think this may be due to a lack of awareness of its value, which may be partly due to a number of persistent misconceptions regarding single-subject studies. In the present paper, we aim to increase the recognition of the value of single-subject studies in the field of psychiatry by discussing some major facts and fictions of single-subject research.

**Figure 2:**
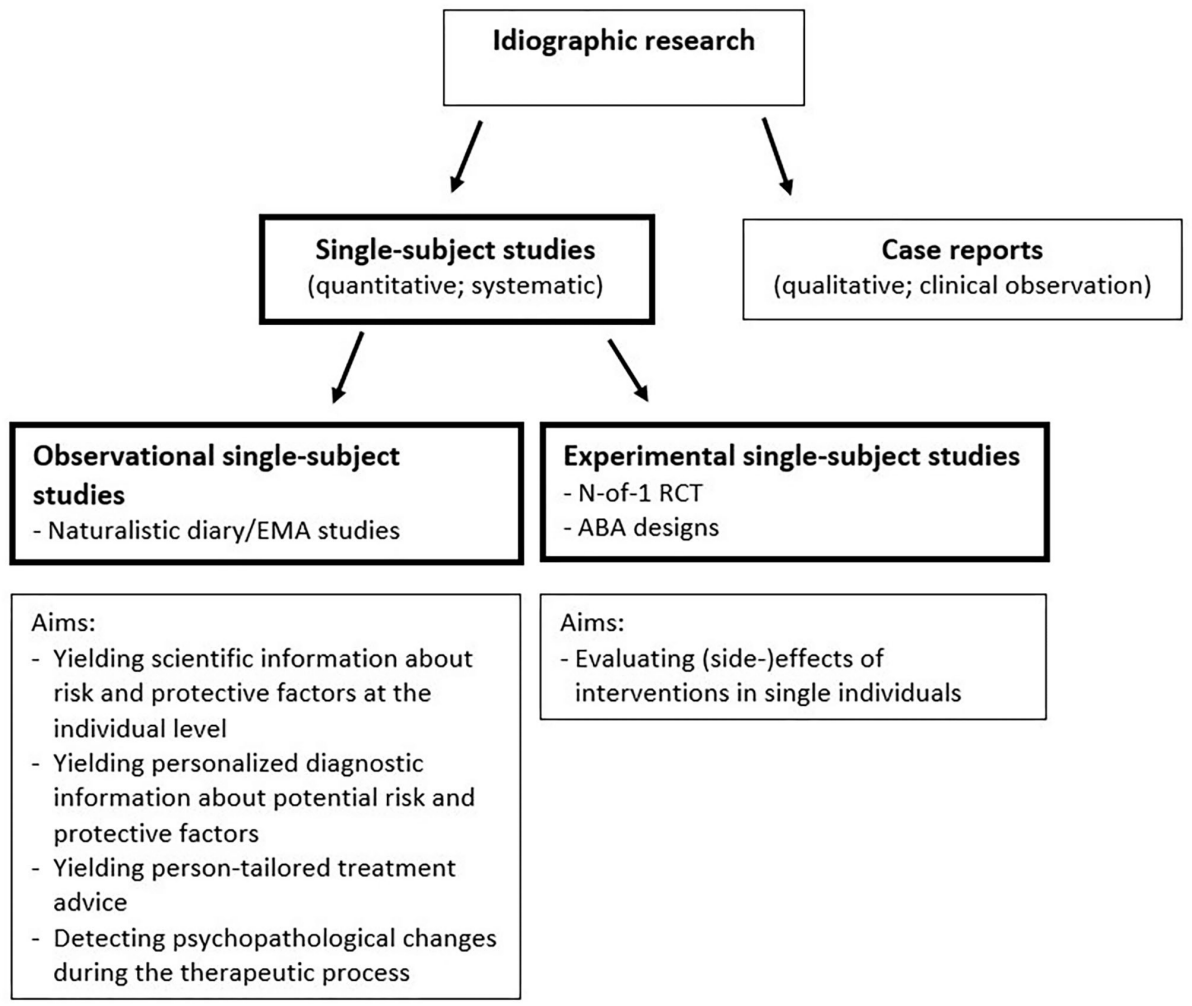
Schematic illustration of different sorts of single-subject studies and some examples of aims of the two types of single-subject studies.

Single-subject studies are characterized by their focus on single persons. This is in contrast to most traditional group-based (“nomothetic”) study designs, which focus on group averages and compare (groups of) individuals with other individuals (such as RCTs, cohort studies or case-control studies). In single-subject studies, data of each individual are analyzed separately and individuals are compared with themselves (13, 14). By virtue of multiple assessments collected within one individual, an individual can serve as his or her own control over time. This allows to quantitatively examine whether changes in one variable are systematically related to changes in another variable within an individual (observational single-subject design), or whether an experimental manipulation is related to a consistent change within this individual (experimental single-subject design; see Figure 2).

More generalizable conclusions can be obtained by replicating multiple single-subject studies on a specific topic. In the presence of great inter- and intra-individual variability, this will only answer questions that apply to individual patients when each participant is analyzed at the intra-individual level. If the same effect is found in a series of single-subject studies, this could potentially be the basis for a generalizable conclusion. That is, the association might be true for the majority of persons [i.e., true in general; (15)]. In case of large heterogeneity, the chance of finding such commonalities for processes underlying psychiatric disorders might not be great. In that case, single-subject studies can be used to link individual-level results to certain person characteristics, or may be used in clinical practice to inform the treatment process.

The single-subject study might be rare nowadays, it has been used much in earlier centuries and has yielded important information about human behavior, physiology and pathology (see Box 1). The use of this design began to decline at the beginning of the 20th century, when people became interested in the improvement of species or races (27). In that time, scientists (and eugenicists) like Pearson and Fisher introduced statistical techniques focusing on group averages, therewith fueling a paradigm shift toward group-aggregated results. This shift toward statistics based on group averages was a logical step to make if the interest is in improving species or plant varieties. For example, if a farmer wants to know which factors improve the growth of lettuce plants, he is not interested in the growth of the individual lettuce plant, but rather in the average yield of the whole field of lettuce plants. However, as we illustrated in the first paragraph of this introduction, many questions in the field of psychiatry apply to individual patients. The almost complete disappearance of the single-subject study at the beginning of the 20th century therefore seems incompatible with the demand for information that applies to individual persons in this field.

Box 1 **The single-subject study has a long history.**

Single-subject research is rather unpopular nowadays, except in some specific subfields of psychology (16). However, it used to be very common. At the end of the 19th century, “the intensive study of individuals” (also called “Idiographic research”) was the most popular scientific approach (15). The term “nomothetic” had a different meaning in those days: nomothetic research was research aimed at establishing general laws and theories (10). It was thought that establishing general laws cannot be done without describing and explaining particular events and individual processes. That is, in order to find out whether something is true for all or the majority of persons, one must first describe and explain what holds for single individuals. Accordingly, nomothetic and idiographic research were seen as complementary. This changed in the beginning of the 20th century. Inspired by famous statisticians and eugenicists like Pearson and Fisher, who introduced techniques like the correlation coefficient and the normal distribution, the research focus shifted from the intensive study of individuals to the study of aggregates from large groups. The label “nomothetic” came to stand for group-based research, and instead of focusing on what is common to all, analyses became focused on what is true “on average” (10, 15).^1^ Although seemingly old-fashioned, several useful findings have sprouted from the study of individuals, for example from quantitative research by Ebbinghaus, Pavlov, Thorndike, Watson, and Shapiro, and qualitative research by Broca, James, Freud, Alzheimer, and Piaget, which has been described elegantly elsewhere (10, 17, 18). One of the most famous adepts of the single-subject approach was Burrhus Skinner, who said that he would rather study one rat for a thousand times than a thousand rats for 1 h each (19). Skinner studied how animal subjects (such as pigeons or rats) acquired certain behaviors in response to stimuli by rewarding or punishing the animal (20). This work on operant conditioning revealed important knowledge of human behavior that is still applied nowadays for addressing clinical problems, such as the treatment of addiction (21), and the development of cognitive behavioral and operant behavioral therapies (22). In medical sciences, single-subject studies are still used occasionally, for example to examine the benefits or side effects of a drug in individual patients. Such “n-of-1 trials” have shown their potential in terms of deciding whether a specific intervention works for a specific individual patient [e.g., (23–26)].

Although they are still relatively rare, in recent years single-subject studies have been more frequently used in the field of psychiatry, possibly due to innovations in ecological momentary assessment (EMA), analytic methods, and technologies (28, 29). Single-subject studies in the field of psychiatry have been applied for several reasons (see Figure 2). First, observational single-subject studies have been done to evaluate temporal associations between variables that may be important in processes underlying psychiatric disorders [e.g., (30–33)]. Such studies can yield very useful information about potential risk and protective factors at the individual level, which can increase scientific as well as clinical insight. Some recent studies have elaborated on this, and used single-subject research for developing personalized diagnostics (34–40) and person-tailored treatment advice (41–44). Another applicability of observational single-subject studies is the examination of the temporal dynamics of single variables, such as the variability or inertia (or autocorrelation, i.e., the degree to which successive observations are related to each other). For instance, Wichers et al. revealed that an increase in autocorrelation in negative affect preceded a relapse of depression in a single patient (45). Thus, single-subject studies can yield information that can be used for detecting psychopathological changes or early warning signals. Another application of the single-subject study is to evaluate the effects and side effects of interventions in single individuals. In the field of psychiatry such experimental single-subject studies have evaluated, for example, the person-specific effects of individualized cognitive therapy for depression in women with metastatic cancer (46), pharmacological treatment for depression (47), stimulants for ADHD in children (48), and treatments for schizophrenia (49). Taken together, single-subject studies are and can be used for different reasons in the field of psychiatry.

Despite a small recent increase in the use of the single-subject study design, it is still relatively scarce in the field of psychiatry. This is remarkable in a field that typically has to deal with a lot of inter-individual heterogeneity and intra-individual variability, and in which there is a high demand for results that apply to individual patients. We believe that this is because of a lack of awareness of the value of single-subject studies rather than a lack of usefulness or feasibility. We will discuss several facts and fictions regarding single-subject research, in order to resolve some existing misconceptions about single-subject studies and make the reader more aware of their value.

## Facts and Fictions

We will now describe several statements that are often heard from, for example, reviewers, members of ethical boards, funding agencies, and colleague researchers. For each statement we will explain whether we think it is a fact or fiction, and elaborate on this.

### Statement 1. Single-Subject Studies Are Case Reports and Therefore Have No Scientific Value

#### Fiction

Although single-subject and case reports both focus on individuals (i.e., are both idiographic; see Figure 2), there are some major differences. A case report is the presentation of an interesting observation on a patient by the treating specialist that lacks a pre-conceived design and systematic assessments. Although case reports may be very informative for generating hypotheses, the lack of systematic design elements makes them prone to bias and invalid inference (14, 50). For instance, a clinician may observe improvement in a depressed patient after a certain therapy [e.g., (51)] and may attribute this improvement to the therapy, while in reality it was due to something else or a spontaneous recovery. Experimental single-subject studies have specific design elements that help elucidate whether the improvement is really due to the therapy. They have a pre-conceived design with different cross-over periods (intervention and control) and pre-planned assessments, often done with validated instruments by an independent researcher [for guidelines see (14, 17, 50, 52)].

Observational single-subject studies are also characterized by a pre-planned design and systematic assessments of outcomes, making them valuable for scientific research and clinical diagnostics. For example, a patient might want to know whether he generally feels more depressed after seeing his mother in law. A single-subject observational study can answer questions about such dynamic associations between variables within one individual, which may go undetected in the clinical care setting.

Thus, if a single-subject study is set up properly and has enough observations to allow for statistical inference, it can yield valid and reliable scientific and clinical evidence (13, 17, 23).

### Statement 2. The Sample Size of Single-Subject Studies Is Too Small to Yield Enough Statistical Power

#### Fiction

An often-heard objection is that the sample size of single-subject studies is too small. However, in single-subject studies time-series data are analyzed for each individual separately. Because of this, the power in single-subject studies depends on the number of repeated observations within a person instead of the number of persons. Thus, if the number of repeated observations within the individual is large enough for the planned statistical analysis, the power is sufficient. A variety of statistical methods exists for the analysis of single-subject data [e.g., (29, 53, 54)].

### Statement 3. The Sample Size of Single-Subject Studies Is Often Too Small to Generalize Findings to the Population

#### Fact

While power of a single-subject study can be sufficient even if *n* = 1 (see Statement 2), the sample size of single-subject studies is still relevant for generalizability to the population. One can only generalize findings to a population if it can be demonstrated that the principle holds in all, or a large majority, of a representative sample, which is not possible if *n* = 1. However, in order to generalize the results of single-subject studies, multiple single-subject studies can be performed in individuals of the same population (direct replication), or in different settings or populations (systematic replication) (55, 56). Results of multiple single-subject studies may subsequently be summarized using for instance meta-analysis (24, 57, 58). If the ultimate goal is to gather scientific evidence concerning questions that apply to individual persons, researchers can build a body of single-case work, eventually leading to a large sample.

### Statement 4. Group-Based Studies Are More Suitable Than Single-Subject Studies to Find Out What Is True in General

#### Fiction

Results from group-based studies yield information about what is “true on average” and typically end up in standardized treatment guidelines. However, in the presence of large inter- and intra-individual differences, average effects are not informative on whether a result is “true in general” (i.e., present in the majority of the sample) (15). For instance, mood disorders are on average associated with higher cortisol levels at the group level (59), but this does not necessarily mean that worse mood is associated with higher cortisol levels in the majority of individuals. A single-subject study repeated in 30 individuals found great individual differences in the within-subject association between mood and cortisol levels (31). Thus, group-aggregated, averaged results are not necessarily or very likely true for each individual in that group. Moreover, the average may also be a poor reflection of what is true for *most* individuals in the group, for example if the distribution of parameters is bimodal or trimodal (60). Also creating subgroups may not solve that problem, because we often do not know by which characteristics we must define subgroups.

More formally, it has been shown that results obtained from group-based studies can only be generalized to individuals when the assumption of “ergodicity” is met. Ergodicity implies that the average, variance, covariance and lagged covariance between variables should be the same for all individuals (homogeneity), and that no changes over time in these statistical characteristics should be present (stationarity) (9, 61, 62). In the absence of ergodicity, effects calculated at the group level, or even at a more homogeneous subgroup level, will not generalize to the individual level (7, 9, 12, 15, 63–65). An association found at the (sub) group level may be weaker, stronger, absent or even reversed in an individual (61). In fact, group-aggregated results may sometimes not even apply to a single individual in that group (10, 15).

### Statement 5. Confounding in Single-Subject Studies Is the Same as in Group-Based Studies

#### Fiction

A point frequently raised by reviewers is that analyses of single-subject studies should be adjusted for relevant demographic or clinical variables. Indeed, these type of variables may confound associations in group-based studies because they may differ between individuals (between-subjects confounding). However, in single-subject studies all variance in the outcome is due to within-person variance in other variables, which may include changes in environmental variables, events, lifestyle- or other behaviors, treatments, etc. (61, 62). Therefore, in single-subject studies variables can only confound an association if they vary within the individual over time (within-subject confounding). Variables that do not show fluctuations over time, such as sex or a stable somatic condition, need (and can) not be adjusted for in single-subject studies. While in group-based studies both between- and within-subjects confounding may occur, in single-subject studies only within-subject confounding may occur. Only if one reverts to a group approach, for example by combining results from multiple single-subject studies, group-level covariates will become applicable again.

### Statement 6. A Single-Subject Study Cannot Establish Causality

#### Fact

It is not possible to establish causality using a single-subject design. Moreover, this is also true for group-based study designs. Hypothetically, the ideal experiment to determine the causal effect of a certain treatment would be to expose an individual to this treatment, observe what happens, and then go back in time and expose the same individual to another condition (i.e., no treatment or placebo), all other things being equal (25, 66). Of course, such a design is not possible as we cannot go back in time. But interestingly, certain forms of single-subject studies come close to this ideal experimental design: the n-of-1 randomized controlled trials (n-of-1 RCTs). In these trials, various conditions (e.g., treatment and placebo, or medicines with varying dosages) are alternated over time and the order of exposure is determined randomly. In this way, each individual serves as his or her own control. The strength of the design increases if multiple cross-over periods are used and patients as well as clinicians and researchers are blinded to the treatment order. A few studies in the field of psychiatry have used such designs successfully to examine the impact of interventions in single patients [e.g., (47–49, 67, 68)]. The deviation of this n-of-1 RCT from the ideal counterfactual experiment is small and concerns only the fact that the individual may have changed over time. In the traditional group-based RCT, groups of people are compared with each other, assuming these groups are similar. This latter assumed similarity is a stronger assumption, and generally not true (69) than the assumption that an individual is similar to him- or herself somewhat earlier in time (66). Furthermore, n-of-1 RCTs often include patients that do not meet the highly selective inclusion criteria of group-based RCTs, and thus provide information for patients for whom there is currently a lack of evidence for treatment efficacy (47). Thus, the n-of-1 RCT appears at least as ideal as the group-based RCT to establish causality, and therefore deserves more attention in the scientific field (25, 66, 70).

In addition to the n-of-1 RCT, other forms of single-subject studies, such as ABA-designs or observational single-subject studies can also contribute information that is important for establishing causality, including strength of the association, consistency of the association in different contexts and times, specificity of the association, temporal precedence, and dose-response relationship (71, 72). Because an individual serves as his or her own control in all single-subject studies, it is possible to determine in a systematic way the strength, consistency, specificity and dose-response relation of an association within that individual. Furthermore, because of the multitude of repeated assessments in single-subject studies, the temporal order of associations can be revealed (30, 31, 73–75). For example, it can be revealed that changes in certain factors systematically precede changes in other variables (i.e., temporal precedence). Even though causality can probably never be completely established, these aspects of single-subject studies are particularly helpful to approach valid causal inference. Also sophisticated approaches to establish causal inference via Directed Acyclic Graphs and Structural Causal models can be applied to single-subject models (76, 77), although these have very strict assumptions that are very hard to meet in practice (77).

### Statement 7. Single-Subject Research Is a Lot of Work

#### Fact

An often-heard statement about single-subject research is that it needs a lot of effort, which is true. Single-subject research involves frequent/repeated assessment during a relatively long period of time, which is time consuming and effortful for both the participant, the researcher, and for therapists if they are involved. However, due to recent technical innovations and increased smartphone and sensor use, collecting ambulatory time-series data has become more feasible. The feasibility has been shown for healthy individuals (37), older adults (78, 79), and also for patients with psychiatric disorders such as severe depression or bipolar disorder (80), panic disorder (36), psychosis (81), eating disorders (82) or ADHD (83). Data collection via a smartphone is more convenient for the participant than paper-and-pencil methods and makes laborious and error-prone data entry unnecessary. The researcher mainly has to focus on data cleaning, statistical analysis and (optionally) feedback generation, for which nowadays more and more automated algorithms are being developed [e.g., (84, 85)].

Single-subject research is also more feasible when participating in a single-subject study is rewarding for participants. For instance, revealing the personal treatment effects or optimal dosage of a certain drug (47), or giving diagnostic information through a personalized feedback report (37, 41, 43) appeared particularly motivating to increase compliance in clinical samples. Feedback reports can contain descriptive feedback [e.g., (41)] or information about potential triggers of symptoms based on statistical models [e.g., (36, 37)]. Although there are still many challenges that need to be resolved (86), personalized feedback may reveal valuable insights for patients and thus help to motivate them to complete the study.

### Statement 8. One Can Just as Well Use Multilevel Modeling in Order to Analyze Data of a Group of Individuals

#### Fiction

In single-subject research, time series of each individual are analyzed separately. But why not use multilevel modeling instead? Multilevel modeling is a well-known tool for analyzing longitudinal data collected in multiple persons, that also allows to study within-person associations. While this is true, multilevel methods still yield group-aggregated results. The fixed effects, which are usually the main outcome of interest, represent the *average* effect in the group. The fixed effects are a mix of within- and between-person effects, but person-mean centering of the predictors can be applied if we are interested in within-person associations[(87); for examples of such studies, see (88–94)]. However, such analyses still yield *average* within-person associations. As discussed in Statement 4, the average may not reflect what is true in general, i.e., for the majority of individuals. If there is large heterogeneity in the sample, for example if the distribution of parameters is bimodal or trimodal, or if the functional form of the model differs across individuals, the fixed effects will be a poor reflection of what holds for individuals (60, 95, 96). As a corollary, also the random effects (the inter-individual differences in the effects) may not be appropriate. Random effects are *post-hoc* estimated deviations from the average effects, and are assumed to be normally distributed around these averages. If the latter are inaccurate, so will be the random effects (60, 95, 96).

Furthermore, while person-mean centering is useful for disaggregating between- and within-person effects of the predictors, with respect to other model features within- and between-person variance is more difficult to separate (for example, the error covariance matrix) (97, 98). Recently, new models like Dynamic Structural Equation Modeling (99), or Bayesian Dynamic Modeling (96, 100, 101) do offer increased possibilities to adequately model other model features within a multilevel framework. However, with increased model complexity, for example with multiple interactions, feedback loops, or non-linear effects, the problem of disaggregating within- and between-person variance in multilevel models becomes quite difficult. Analyzing data at the individual level leaves more room for modeling such complexities (56, 95, 96).

Nevertheless, there are situations in which multilevel modeling is the preferred statistical method. Multilevel models have the advantage that they can “borrow strength” from the data of other individuals (95, 101). This may be a great advantage if individual time-series data are noisy, the number of repeated measures is low, or the sample is homogeneous. In such cases, multilevel models will yield better estimates than single-subject analyses. A replicated single-subjects approach may be the statistical method of choice in case of large heterogeneity, many repeated observations, or high complexity (33, 56, 60, 95, 96, 101).

## Discussion

Despite the relatively high demand for information applying to individual persons, the single-subject study is not often used in the field of psychiatry. We believe that this is because of a lack of awareness of their value rather than a lack of usefulness or feasibility. In the present paper we aimed to resolve some common misconceptions and beliefs about single-subject studies by discussing some commonly heard facts and fictions.

Single-subject studies can be particularly useful and have additional value in several situations. For example, when there are large inter-individual differences in the processes under study, when these processes are very complex or nonlinear, or can change over time. Additionally, single-subject studies have the advantage that they may include any patient (also those with complex or rare diseases), and (in the case of experimental single-subject studies) are able to adjust the treatment when deemed necessary (102, 103). This increases the ecological validity of the single-subject study, and makes it particularly useful in situations when large-group studies are not feasible; for instance because the disease or event under study is rare, the patient is complex (e.g., many comorbidities), the setting is complex (e.g., palliative care), the intervention is highly expensive or controversial, or the intervention contains person-specific elements (46, 56, 102, 104, 105). Furthermore, single-subject studies may involve patients more in their treatment process, thereby increasing patient empowerment and shared decision making (68). The single-subject study may also contribute in the diagnostic process, to evaluate factors contributing to treatment responses, or to evaluate efficacy of certain treatments (34, 38–41, 43, 44). Practically, this has led to the recent development of algorithms and clinical care applications that implement single-subject analyses in the diagnostic process in clinical care settings [(42, 44, 106) *conference abstract*]. Single-subject studies can also be used to obtain a detailed description of a particular approach applied to an individual in order to test an existing clinical theory [theory exemplification; (107)]. While group-based studies often only study a limited number of aspects belonging to a theory, a single-subject study can map in detail all its aspects together in one person. For example, a single-subject study could detail all processes underlying response to cognitive therapy in a specific patient [e.g. (108)], thereby showing how to optimally apply an existing theory underlying cognitive therapy. Complex statistical models can be linked to processes underlying psychiatric disorders [for example how a panic attack evolves in a specific patient, (109)], which may help in understanding their mechanism in individual patients.

Despite these advantages of single-subject studies, group-based studies are more appropriate if one wants to make inferences about average tendencies in the population, such as the prevalence, incidence or average risk of a disorder, or the average effect of certain treatments in the whole population or a certain subpopulation. For instance, if one wants to know whether legalizing cannabis helps in reducing the prevalence or incidence of psychotic disorders in the population. Moreover, group-based studies are more appropriate if individual time-series data are very short or noisy, or the process under study is homogeneous across individuals (33, 95, 101). Thus, group-based and single-subject studies can both be useful and are appropriate in different situations.

In some circumstances, the group-based and single-subject approach may be combined. A first reason for combining these designs is to identify commonalities across persons, in order to increase generalizability to the population. Practically this can be done by combining group- and individual-level analyses in one model, for example using Group Iterative Multiple Model Estimation [GIMME (60)], or meta-analytical techniques for pooling data from multiple single-subject studies (57, 58, 110). Related to this, pooling data from multiple single-subject studies can be used to link individual-level results to certain between-subjects characteristics. For instance, results from multiple experimental single-subject studies may be pooled and linked to patient characteristics in order to identify which patient characteristics are associated with better outcomes of a certain treatment (103). Likewise, data from multiple observational single-subject studies may be pooled in order to identify whether person-specific associations between variables can be linked to certain patient characteristics such as the presence of a depressive disorder [e.g. (31, 73, 111)], or different severity of depressive symptoms (112). Another reason to combine the single-subject with the group-based approach would be to examine the group-level effectiveness of an individualized treatment or lifestyle advice that is based on single-subject analyses of diary observations [e.g., (43, 44)]. In this way, the single-subject study design can be of added value to the increasing urge for personalized patient care in mental health care settings (29, 113–115).

## Conclusion

In the field of psychiatry, single-subject studies are still relatively scarce. We hope that we have resolved some misunderstandings surrounding single-subject studies and have increased the reader's awareness of possibilities and impossibilities of the single-subject design. Although single-subject studies are definitely not suitable in all circumstances, we believe that they deserve more attention in the field of psychiatry, especially in view of the current urge for personalized patient care, increased importance of shared decision making, increased availability of electronic devices and sensors, and recent advancements in analytic methods for time-series data.

## Author Contributions

MZ, HR, ES, SB, MW, and EB: conceptualization, review, and editing. ES, SB, and MW wrote sections of the manuscript. MZ, HR, and EB: wrote first draft of major parts of the manuscript. All authors contributed to the article and approved the submitted version.

## Conflict of Interest

The authors declare that the research was conducted in the absence of any commercial or financial relationships that could be construed as a potential conflict of interest.

## Footnotes

^1^In view of the ambiguous meaning of the word “nomothetic,” we use the term “group-based” throughout the paper when we refer to a study that focuses on group-aggregated results.
